# Utility Evaluation of Porcine Enteroids as PDCoV Infection Model *in vitro*

**DOI:** 10.3389/fmicb.2020.00821

**Published:** 2020-04-23

**Authors:** Hao Luo, Jingyou Zheng, Yunlu Chen, Tingjun Wang, Zhenning Zhang, Ying Shan, Jidong Xu, Min Yue, Weihuan Fang, Xiaoliang Li

**Affiliations:** Zhejiang Provincial Key Lab of Preventive Veterinary Medicine, Institute of Preventive Veterinary Medicine, College of Animal Sciences, Zhejiang University, Hangzhou, China

**Keywords:** porcine enteroids, crypts, porcine deltacoronavirus, infection model, differentiation

## Abstract

Porcine deltacoronavirus (PDCoV) is a novel emerging enteric coronavirus found in pigs. Intestinal enteroids, which partially recreate the structure and function of intestinal villi-crypts, have many physiological similarities to the intestinal tissues *in vivo*. Enteroids exhibit advantages in studying the interactions between intestines and enteric pathogens. To create a novel infection model for PDCoV, we developed an *in vitro* system to generate porcine intestinal enteroids from crypts of duodenum, jejunum, and ileum of pigs. Enterocytes, enteroendocrine cells, Paneth cells, stem cells, proliferating cells, and goblet cells were found in the differentiated enteroids. Replication of PDCoV was detected in the cultured enteroids by immunofluorescence and quantitative RT-PCR. Double immunofluorescence labeling demonstrated that PDCoV was present in Sox9-positive intestinal cells and Villin1-positive enterocytes. There were multiple cellular responses shown as changes of transcription of genes related to mucosal immunity, antiviral genes, and marker genes of stem cells and other cells in the enteroids infected with PDCoV. We conclude that the 2-D enteroids derived from porcine jejunum can be used as an *in vitro* multicellular model for the investigation of pathogenesis and host immune responses to porcine enteric pathogens, such as PDCoV.

## Introduction

Porcine enteroviruses are a group of highly contagious pathogens that cause serious economic losses worldwide. The porcine intestinal epithelial cell lines SD-PJEC (Sun et al., 2012) and IPEC-J2 (Jung et al., 2018) are most commonly used for *in vitro* studies and characterization of the interaction between intestinal cells and enteroviruses. However, these cell lines consist of only a single type of cells, lacking the complexity and physiology of multiple cells in the native intestinal tracts. These cell lines are prone to spontaneous mutation upon repeated passages, reducing their applicability (Yin et al., 2019). The eventual outcomes of viral infection are generally complicated and associated with multifaceted host responses to different viruses. Hence, more representative models comprising multiple cells mimicking the intestinal tracts are required for *in vitro* studies.

In 2013, researchers successfully isolated the porcine enteroids for the first time (Gonzalez et al., 2013). Enteroids display more advantages when compared with the transformed or immortalized cell lines. The enteroids contain multiple distinct intestinal cell types, such as entero-endocrine cells, enterocytes, goblet cells, and stem/progenitor cells, and mimic the villus and crypt domains comparable to the architectures of the intestine *in vivo* (Gonzalez et al., 2013; Sato and Clevers, 2013; Powell and Behnke, 2017). Moreover, they could be passaged and banked for future use like the cell lines (Powell and Behnke, 2017). A recent study indicates that an *in vitro* 2D gut model could be established to expose the luminal side of the intestine to microorganisms, chemicals, and biologically active and toxic compounds, making it feasible to study innate immunity to pathogen infection and disease-associated polymorphisms (van der Hee et al., 2018). Porcine enteroids are also reported to be more amenable for lentiviruses transduction (Khalil et al., 2016) and plasmid transfection. This provides a robust tool to study intestinal diseases. Using intestinal enteroids as a model system, porcine epidemic diarrhea virus (PEDV) was found to infect multiple lineages of porcine intestinal epithelial cells, providing insights into enteric virus-targeted intestinal cell populations (Li et al., 2018). Although porcine intestinal enteroids display a great potential as an *in vitro* host-virus interaction model, limited information is available on the possibility of its continuous uses in *in vitro* cultures, susceptibility to infection with porcine deltacoronavirus (PDCoV), and the types of immune responses induced by viral infections.

Porcine deltacoronavirus is an enveloped, single-stranded, positive-sense RNA virus containing a genome approximately 25 kb in length and belonging to the family Coronaviridae (Homwong et al., 2016). The virus was initially isolated from the fecal samples of pigs in 2009 in Hong Kong. However, its etiological role was not identified until 2014 when it was found to cause diarrhea in pigs in the United States (Woo et al., 2012; Wang et al., 2014). To date, PDCoV has been reported in countries like the United States, Canada, South Korea, China, Thailand, Laos, and Vietnam (Homwong et al., 2016; Janetanakit et al., 2016; Jang et al., 2017; Saeng-chuto et al., 2017; Ajayi et al., 2018; Le et al., 2018). PDCoV, as a newly identified swine entero-pathogen, warrants further study because there is a paucity of information on host responses to its infection and pathogenesis (Koonpaew et al., 2019).

In this study, the three-dimensional enteroids from porcine duodenum, jejunum, or ileum were obtained and converted into monolayer porcine intestinal enteroids (MPIEs). PDCoV was able to infect the differentiated MPIEs and induce multiple cellular responses. This novel *in vitro* platform could be employed to study the molecular mechanisms of PDCoV infection.

## Materials and Methods

### Isolation of Porcine Small Intestinal Crypts

The study was carried out according to the guidelines of the Animal Care and Use Committee of Zhejiang University (Approval No. 14820). Three-week-old healthy Yorkshire piglets were used for isolation of crypts from anterior duodenum, jejunum, and ileum in accordance to a previous protocol with some modifications (Sato et al., 2011; Mahe et al., 2015). QPCR and RT-PCR were performed to detect the existence of different pathogens, such as mycoplasma, PEDV, PDCoV, Porcine Reproductive and Respiratory Syndrome Virus (PRRSV), Porcine Circovirus type 2 (PCV2), and Pseudorabies Virus (PRV), then we discarded the positive samples to ensure no contamination in the cultured enteroids.

### Culture of Porcine Intestinal Enteroids (PIEs)

For three-dimensional PIE culturing, approximately 50 crypts were mixed with 20 μL of matrigel (Corning, United States) and seeded in a 48-well plate pre-warmed at 37°C. After solidification of the matrigel (about 10 min), 250 μL of Porcine Intestinal Proliferation Medium (PIPM) (Shanghai Biomed 121 Co., Ltd) was added and incubated at 37°C and 5% CO_2_. The medium was changed every other day. For passage of the enteroids, 1 mL of cold DPBS (Dulbecco’s Phosphate Buffered Saline) was added after the medium was removed. The enteroids were subjected to repeated and vigorous pipetting into small pieces using a 1-mL pipettor. The pieces were pelleted by centrifuging at 200 × *g* for 5 min at 4°C and then mixed with matrigel for plating.

Culture of the MPIEs was carried out as recommended (Ettayebi et al., 2016; Thorne et al., 2018; van der Hee et al., 2018). Highly confluent enteroids were obtained after 7 days culture and dissociated into a single-cell suspension with EDTA-trypsin (Invitrogen, United States). About 4 × 10^4^ cells were seeded into a 96-well plate coated with 2.5% matrigel. The cells were cultured in PIPM for 24 h at 37°C and 5% CO_2_ and the medium was replaced with 250 μL of Porcine Intestinal Differentiation Medium (PIDM) and refreshed every other day.

### Virus, Cell Lines and Antibodies

The PDCoV strain ZJ17DQ0301 (GeneBank accession number: MF461406) was isolated from a clinically diseased pig in Zhejiang, China (Shan et al., 2018). The IPEC-J2 cell line was cultured at 37°C and 5% CO_2_ in Dulbecco’s Modified Eagle Medium: Nutrient Mixture F-12 (DMEM/F-12) (Gibco, United States) supplemented with 10% fetal bovine serum (FBS) (Yeasen, China), 100 U/mL penicillin, 100 μg/mL streptomycin, and 0.25 μg/mL amphozone (Gibco, United States). The virus was propagated for 20 passages, titrated in IPEC-J2 cells, and stored at −80°C. Mouse monoclonal anti-PDCoV N antibody (2H7) was raised in our laboratory.

### Virus Infection

The MPIEs were allowed to differentiate for 3 days and IPEC-J2 cells at 80% confluency were used. The cells were washed thrice with DMEM (Gibco, United States) before PDCoV infection. The inoculated cells were incubated for 2 h at 37°C and 5% CO_2_ to allow virus attachment. The cells were again washed twice with DMEM and cultured in DMEM containing 8 μg/mL of trypsin. PDCoV at MOI of 0.1 and 1 was used for respective experiments on viral growth and cellular responses. The cells cultured in each well were separately collected at different times (Figure 5A) after viral infection for total RNA extraction, or directly used for cell staining.

### Reverse Transcription Quantitative PCR (RT-qPCR)

Total RNA from cell samples in each well was separately extracted using the RNA Extraction Kit (Biotek, RP4002). Purity and concentration of the RNA samples were measured using NanoDrop (Thermo Fisher). Total RNA was used to synthesize cDNA with HiScript^®^ 1st Strand cDNA Synthesis Kit (Vazyme, R211-02) according to the manufacture’s protocol.

For qPCR, the HiScript One Step qRT-PCR Probe Kit (Vazyme, Q222-01) was used to detect the viral RNA copies, and SYBR Green Kit was used to analyze expression of cellular genes on a Mx3005P Real-Time PCR system (Agilent, United States). Gene-specific primers are listed in Table 1. The cycling parameters included initial denaturation at 95°C for 30 s, followed by 45 cycles (5 s at 95°C and 40 s at 60°C), with a final cooling at 42°C for 30 s. The viral RNA copies were calculated as described (Zhou et al., 2019). The qPCR parameters for quantification of relative expression of cellular genes were: 95°C for 5 min for initial denaturation; 40 cycles of 95°C for 10 s, 60°C for 30 s, followed by a dissociation curve segment (95°C, 1 min; 60°C, 30 s; 95°C, 30 s). The relative expression of the host cell genes was normalized to GAPDH.

**TABLE 1:** Primer sequences used in this study.

Gene Name	Forward	Reverse	Amplicon size (bp)	Annealing temp (°C)
Mucin2	ATCTACACCAAGGTCTATTCCCGAGCTGGC	GGCTTGTTGATCTTCTGCATGTTCCCGAAC	209	60
MMP9	CAGTATCTGTATGGTTCTCGCCCTAAACCTG	GCCAGCAGTGGAGCGCTCAG	141	60
Ex-FABP	GAGGAATGCAGCGTTGATGAAATG	GAAAGTTACTGTTTATTTGGGCAAAAAGGAG	177	60
IL-8	ACTTCCAAACTGGCTGTTGCCTTCTTG	GGTCCACTCTCAATCACTCTCAGTTCCTTG	248	60
IL-1 beta	GAGCATCAGGCAGATGGTGTCTGTC	GGAGTTTGCACTCCATAGACTGCACG	194	60
CXCL11	GAAGGGCATGGCTATAGTCTTGGCTGTC	CAGGGTGACAATCACTTCTGTTTTGTCACAG	190	60
CXCL3	CTGCAGACGGTGCAAGGAATTCACCTC	GATTTTCTTAACCATGGGGGCTTCAGGGTTG	153	60
IL-28b	CCTTCAAGAGGGCCAAGGATGCC	GTGAAGGGGCTGGTCCAGGC	209	60
IL-29	ATGGCTACAGCTTGGATCGTGGTG	GAGGGGAGAGCTGCAGCTCC	216	60
OAS1	TTCATCGAAGACCACCTCCTGCCAAACAC	CAGCATCTGATCGGCCCCTGAGGGTC	187	60
OAS2	CCTCTAGAGGCCATCAGGCAGTTGC	AGCATGGTTGACAAGACCAGGGCAC	216	60
HERC5	GGCACTGTAAAGAGATGGCTTGCTGATGTGG	CGGTTATCGTGTTGGTGATGCAGTCCTTCC	220	60
ISG15	GACTGCATGATGGCATCGGACCTGAAG	GGTCTCTGAAGCTTTGCACCATCAACAG	178	60
Lysozyme	TATGATCGGTGCGAGTTCGCCAGAATTCTG	GTGTCTTGCCATCATTACACCAGTAGCGGC	199	60
FZD5	CATCTTCACGCTGCTCTACACGGTGC	GACCAAATCCAGACGCCTGACGTGATG	216	60
Lgr5	GAACAGCGAGCCTGGAGAGTCTGACTTTAAC	TGGCAAGGTGGAAAATGCATTGGGGTGAATAG	290	60
Bmi1	CCTGGAGAAGGAATGGGCCACTTC	GAGGATGAGGAGACTGCACTGGAGTGC	213	60
ChgA	GAAAGAGCTCCAAGACCTCGCTCTCCAAG	AGAATCTCCTCTTTTTTCCGCAGCCTCCTTG	181	60
TFF3	GGCTCATCGCTTCTGCTTTTCTCTCACCTG	AAGGGAGCGAGCATGACTCGCAG	220	60
Sglt	GATTACATCCAGTCTGTCACCAGTTACTTGGGACC	AGTGCACACCACAGATAATTGTGGGACAGTTG	217	60
L-Fabp	ATACCAAGTACAGAGCCAGGAAAACTTTGAGGC	TCCCCAGTCATGGTCTCCATCTCACAC	213	60
GAPDH	CAAAATATGATGACATCAAGAAGGTGGTGAAGCAGGC	AGGAAATGAGCTTGACGAAGTGGTCGTTGAG	180	60
STAT1	GCGGCAGAATTCCGACACCTGCAAC	AGCTGGCTGACGTTGGAGATCACCAC	191	60
STAT3	CCAACATCTGCCTGGATCGGCTAG	CTGAACAGCTCCACGATTCTCTCCTC	175	60
Sox9	GACCTGAAGAAGGAGAGCGAAGAGGACAAGTTCC	TCTCATTCAGCAGTCTCCAGAGTTTGCCCAGAG	271	60
Ki67	GGCACATGCCATTTAAAAGGCCGTCG	CTGGAATTTCGAGGACCAGTGAATGAAGGC	217	60

### EdU Assay

The EdU assay was used for detection of PIEs proliferation. The PIEs were treated for 1 h at 37°C with EdU (Beyotime, China) at a final concentration of 10 μM. The medium was removed, and the cells were fixed in 4% paraformaldehyde at RT for 30 min and permeabilized with 1% Triton X-100 for 20 min. The cells were then stained according to the manufacturer’s protocols and imaged by a fluorescence microscope X81 (Olympus, Japan). Image synthesis, processing, and cell counting were performed using Fiji ImageJ (Schindelin et al., 2012). Proliferation was described as the growth rate, represented as the number of EdU + cells divided by the number of DAPI + cells.

### Periodic Acid Schiff (PAS) Staining Assay

The presence of goblet cells in monolayer PIEs was observed by PAS staining assay according to the manufacturer’s protocol. First, the cell samples were oxidized in 0.5% periodic acid solution for 10 min at RT followed by incubation in Schiff solution (Servicebio, China) for 15 min. Thereafter, the cells were counterstained in Mayer’s hematoxylin for 1 min. The images were captured using a microscope X81 (Olympus, Japan).

### Indirect Immunofluorescence Assay

Cell samples were fixed with 4% paraformaldehyde for 15 min and permeabilized by 1% Triton X-100 for 10 min at RT. The cells were probed overnight at 4°C with relevant antibodies prepared in PBS (10 mM, pH 7.4): antibodies against Lysozyme (ab74666, Abcam, United Kingdom), Chromogranin A (20086, ImmunoStar, United States), Villin1 (1D2C3, Dako, Denmark), E-cadherin (NCH-38, Dako, Denmark), Sox9 (EPR14335, Abcam, United Kingdom), and monoclonal antibodies against PDCoV N proteins (2H7). Secondary antibodies were either Alexa 488- or Alexa 596-conjugated (ThermoFisher Scientific, United States). Nuclei were stained with DAPI (1 μg/mL). The stained cells were examined by the inverted fluorescence microscope X81.

### Flow Cytometry

To measure the frequencies of CD44+ stem cells in MPIEs, the cell samples were harvested by centrifuging for 5 min at 200 × *g*. The cells were fixed and permeabilized as above and stained, after two washes with PBS, with 1 μg of FITC mouse anti-human CD44 (Thermo, 11-0441-81, clone: IM7) and 1 μg of PE mouse anti-human CD326 (BD, 565399, clone: EBA-1) for 30 min in the dark at RT. The cells were then washed three times with PBS, centrifuged at 400 × *g* for 5 min each, resuspended in 500 μL of PBS, and subjected to flow cytometry (ThermoFisher, United States). A total of 2 × 10^4^ gated events were counted for each sample. The frequencies of stem cells were represented as a ratio of the CD44 + cells to the total CD326 + cells. IgG isotype of FITC or PE was used as a negative control.

### Statistics

All experiments were conducted in triplicates. The data were analyzed by the Student’s *t*-test using GraphPad Prism7 (GraphPad Software, Inc.) and the results are presented as mean ± standard deviation (SD).

## Results

### Three-Dimensional Porcine Enteroids Culture

**FIGURE 1:**
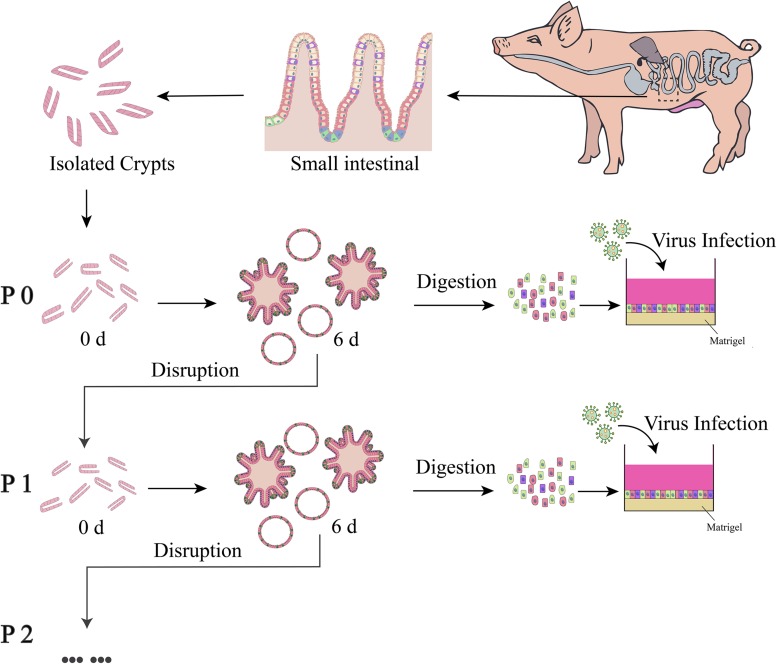
Schematic diagram of culture, passaging and usage of enteroids. The enteroids were cultured for about 6 days and then disrupted by scraping and repeated pipetting. The dispersed enteroids were used for passaging or preparation of a single-cell mixture to be seeded in a 96-well plate pre-coated with 2.5% Matrigel.

**FIGURE 2:**
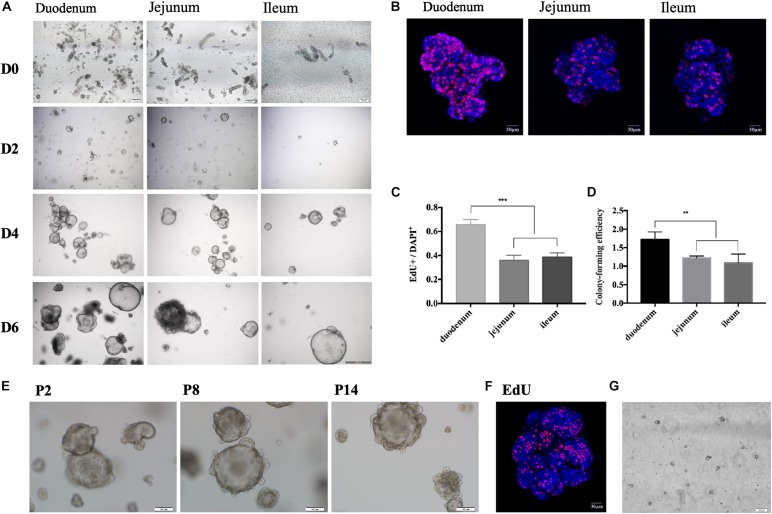
Growing 3-D porcine intestinal enteroids. **(A)** The time course of development of undifferentiated porcine duodenum **(left)**, jejunum **(middle)**, and ileum **(right)** enteroids. (Scale bar = 100 μm). **(B)** EdU staining of the enteroids from duodenum, jejunal, and ileum after 6-day growth (Scale bar = 5 μm). **(C)** The proportion of EdU positive cells in duodenum (*n* = 10), jejunum (*n* = 12), and ileum (*n* = 9) enteroids. **(D)** Comparison of the colony-forming efficiency at day 7 of the enteroids from duodenum, jejunum, and ileum. **(E)** Representative morphological images of jejunal enteroids under different generations (Scale bar = 100 μm). **(F)** The 14th generation enteroids freshly stained with EdU (red) (Scale bar = 100 μm). **(G)** Morphology of jejunal enteroids at day 2 after cell resuscitation (Scale bar = 100 μm). (*p < 0.05, **p < 0.01, and ***p < 0.001).

The protocol of *in vitro* culture and passage of porcine enteroids is shown in Figure 1. We isolated crypts from the duodenum of 3-week-old piglets first and then from jejunum and ileum (*n* ≥ 3). We found it easier to harvest crypts from duodenum than from jejunum or ileum. All cultured crypts from three intestinal segments could gradually differentiate into budding spherical enteroids from small round cell clusters (Figure 2A). The growth rate of the enteroids from the duodenum (*n* = 4) was significantly higher than those from the jejunum and ileum (*p* < 0.001) (Figures 2B,C). The colony-forming efficiency (CFE) of the enteroids derived from duodenum was also significantly higher than those from jejunum and ileum (*p* < 0.001) (Figure 2D). The enteroids were reported to be continuously passaged *in vitro* (Sato et al., 2011; Shaffiey et al., 2016). In our experiments, the jejunal enteroids were passaged more than 14 times (P14) and there were no morphological changes even after 14 passages, as compared with earlier generations (Figure 2E). The growth rate of the P14 enteroids did not decrease as compared with that of the passage 1 (P1) enteroids (Figure 2F). The jejunal enteroids frozen in liquid nitrogen for 1 month were still culturable and could grow to multicellular spheroids again (Figure 2G). In summary, the three-dimensional (3-D) porcine enteroids could be generated using the crypts from duodenum, jejunum, and ileum.

### Monolayer Porcine Intestinal Enteroids (MPIEs)

**FIGURE 3:**
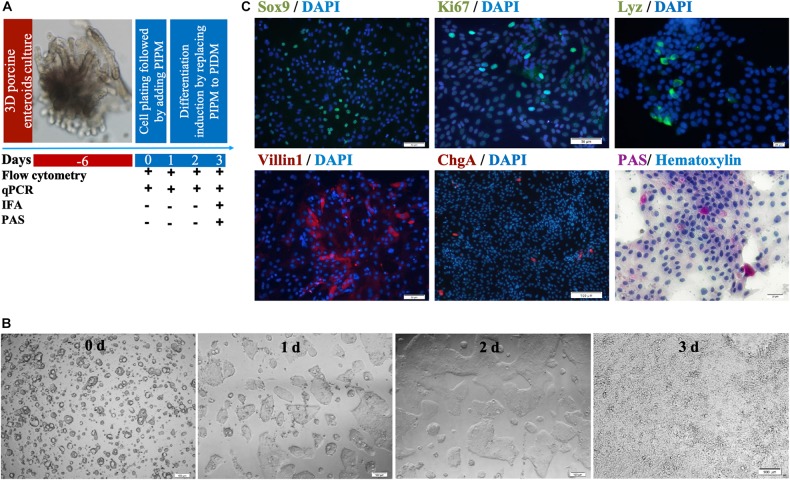
Identification of cell subsets in differentiating MPIEs. **(A)** The schedule of sample collection of the differentiating two-dimensional porcine enteroids. **(B)** Representative images of monolayer enteroids at different time points (Scale bar = 100 μm). **(C)** Identification of cell subsets in differentiating enteroids. Transcription factors Sox9 expressed in CBC stem cells (green) (Scale bar = 50 μm), *Ki67* protein expressed in TA proliferating cells (Scale bar = 50 μm), Lysozyme (Lyz)-containing Paneth cells (green) (scale bar = 20 μm), apical brush border protein Villin1 expressed in enterocytes (green) (Scale bar = 50 μm), Chromogranin A (ChgA)-containing enteroendocrine cells (red) (Scale bar = 100 μm) and Periodic acid-Schiff stain-reacting goblet cells (purple), nuclear (blue).

Three-dimensional enteroids have been used as virus infection models by microinjection (Saxena et al., 2016; Wilson et al., 2017; Holly and Smith, 2018). Here we show that a two-dimensional (2-D) enteroid could be easily infected with PDCoV as a model virus via conventional inoculation as well. We designed our experimental scheme to culture the 2-D porcine enteroid epithelial cell monolayers (Figure 3A) and identified the cell repertoires. We seeded the trypsin-treated round cell cluster in 96-well plates coated with 0.25% matrigel. The cells formed small round cell clusters, and then grew into a tight epithelial layer within 3 days (Figure 3B). IFA and PAS staining were used to examine the abundance of different cell subpopulations at the third passage post-differentiation (Figure 3C). Immunofluorescence of Sox9 and Ki67, recognized as the marker of stem/progenitor cells and TA proliferating cells, respectively, was shown over the 2-D enteroid monolayers. The presence of enterocytes was confirmed by immunostaining the apical brush border protein Villin1. Goblet (PAS+) and enteroendocrine cells (ChgA+) could be found in the MPIEs, but at low levels. Lysosome protein (Lyz) was used to identify porcine Paneth cells.

**FIGURE 4:**
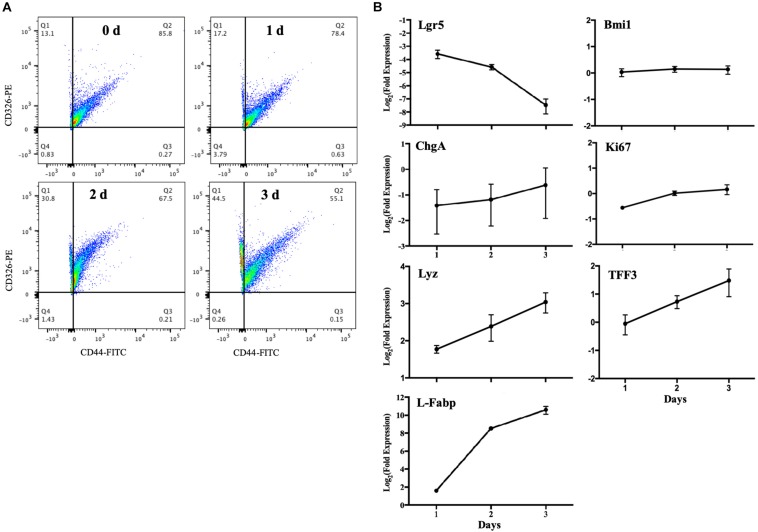
Changes in cellular sub-populations in differentiating MPIEs. **(A)** CD326 (Epcam) versus CD44 expression in cells harvested at different time points. **(B)** Transcriptional changes of the major cell marker genes in 3 days of differentiation detected by RT-qPCR. The transcription levels were normalized to that of *GAPDH* and the relative fold changes were calculated using the 2^–ΔΔCT^. Each bar indicated means ± standard deviations for three independent trials. The abbreviations on the map are leucine-rich-repeat-containing G-protein-coupled receptor 5 (*Lgr5*), B cell-specific moloney murine leukemia virus integration site 1 (*Bmi1*), Ki-67 (*Ki67*), chromogranin A (*ChgA*), trefoil factor 3 (*TFF3*), lysozyme (*Lyz*) and fatty acid-binding protein 1 (*L-Fabp*).

It was reported that murine crypts expressed CD44 only, and specific expression of CD44 was strongly correlated with the LGR5 and HOPX positive cells; CD326 (Epcam) expression, however, could be detected in all kinds of epithelial cells (Gracz et al., 2013). Another study showed that CD44 marker could be applied for sorting intestinal epithelial stem cells by FACS (Chang et al., 2016). Thus, we used CD326 (Epcam) to detect epithelial cells and CD44 to detect intestinal epithelial stem cells. The flow cytometric data revealed that the frequency of intestinal stem cells (CD326+ and CD44+) decreased from 85.8 to 55.1% after MPIEs differentiation from Day 0 to Day 3 (Figure 4A). The relative expression level of the stem cell marker gene *Lgr5* decreased over 10-folds from Day 1 to Day 3, while the transcriptional levels of marker molecules of epithelial cells, such as entero-endocrine cell (*ChgA*), goblet cells (*TFF3*), enterocytes (*L-Fabp*), and Paneth cells (*Lyz*) were upregulated to different degrees (Figure 4B). No distinct changes were seen in the transcription of the + 4 stem cell marker gene, *Bmi1*, and proliferation antigen Ki67-related gene (Figure 4B). Collectively, these results indicate that the MPIEs contain multiple intestinal epithelial cell lineages.

### PDCoV Replication in MPIEs and Its Localization

**FIGURE 5:**
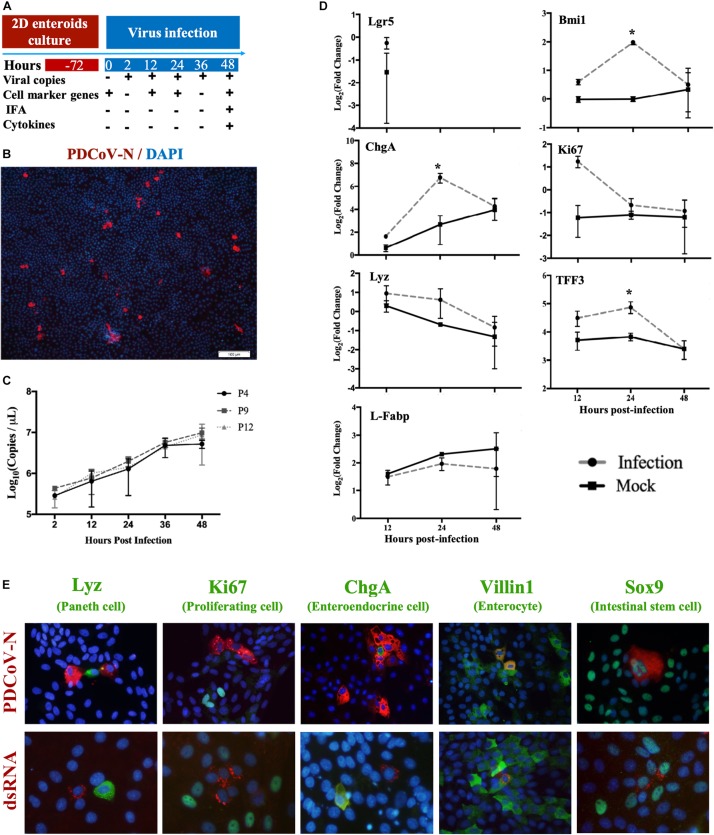
Infection of MPIEs with PDCoV. **(A)** The schedule of sample collection after infection of MPIEs by PDCoV. **(B)** Immunostaining of MPIEs infected with PDCoV for 48 hs. PDCoV is shown in red using the antibody against viral structural protein PDCoV-N. DAPI-stained nuclei are shown in blue (Scale bar = 100 μm). **(C)** Viral genome copies detected in MPIEs at different times of PDCoV infection (2, 12, 24, 36, and 48 h) by RT-qPCR (*n* = 3, mean ± SD). **(D)** The transcriptional changes of the selected marker genes of the infected cells at 12, 24, and 48 h normalized to 0 h controls. Mock-infected cells were used as controls. (*p < 0.05). **(E)** Double immunofluorescence labeling of the jejunal MPIEs after 48 h of infection with PDCoV. Paneth cells, proliferating cells, entero-endocrine cells, enterocytes, and intestinal stem cells were labeled with Lyz, Ki67, ChgA, Villin1, and Sox9 (green), respectively. PDCoV was labeled with anti-PDCoV N protein antibody (red). DAPI was used for nuclear staining.

Based on the MPIEs culture system established as above, we investigated PDCoV replication and localization in the MPIEs according to an infection scheme (Figure 5A). By immunofluorescence, we found that the PDCoV N protein was located in the cytoplasm (Figure 5B). In passage 4 cells inoculated with PDCoV, viral RNA copies at 48 h increased 76.4-folds compared with that of 2 h (Figure 5C). PDCoV could replicate similarly in different MPIE passages (P4, P9, and P12) (Figure 5C). There was apparent co-localization of the PDCoV N protein with Sox9 or Villin1 by using double-immunofluorescence labeling but not with Ki67, ChgA, or Lyz (Figure 5E). These results indicate that PDCoV could have tropism for stem/progenitor cells and enterocytes in porcine enteroids.

### Transcriptional Changes of Cell Subpopulation Marker Genes in MPIEs Infected by PDCoV

The above results show that several cell lineages could be infected by PDCoV. We analyzed transcription of the sub-population cell markers in response to PDCoV inoculation in order to study the influence of PDCoV infection on MPIEs differentiation. The results showed that expression of the stem cell marker gene *Lgr5* was not detectable 24 hours post-infection (hpi) (Figure 5D). The transcription level of other marker genes, *Bmi1* (+4 stem cell), *Ki67* (TA cell), *ChgA* (Entero-endocrine cell), *Lyz* (Paneth cell), and *TFF3* (Globet cell), in PDCoV-infected MPIEs were higher than that in mock-infected cells (Figure 5E). Transcription of *Bmi1*, *ChgA*, and *TFF3* was upregulated at 24 hpi (*p* < 0.05) (Figure 5E). However, transcription of *Ki67* was higher than mock at 24 and 48 hpi (Figure 5E).

### Comparison of Immune Responses Between MPIEs and IPEC-J2 Upon Infection by PDCoV

**FIGURE 6:**
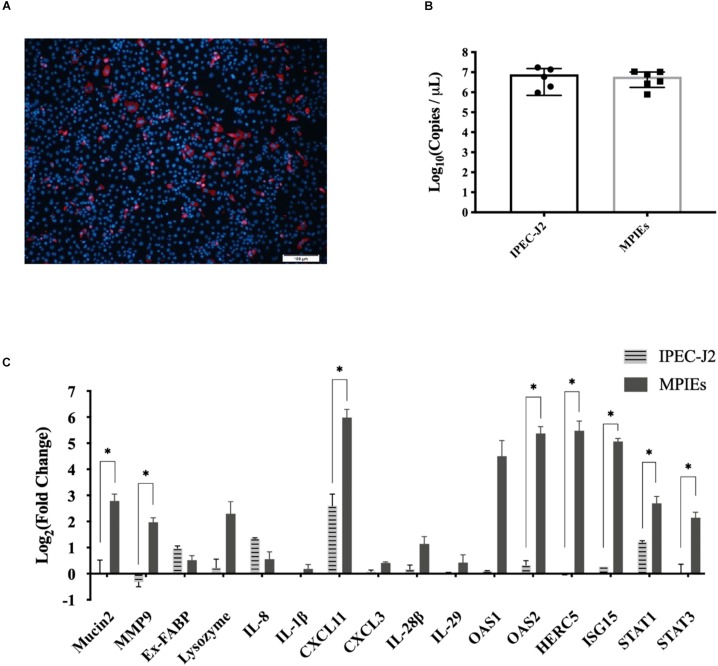
Comparison of innate immune responses of IPEC-J2 and MPIEs to PDCoV infection. **(A)** Immunostaining of IPEC-J2 cells infected with PDCoV for 48 h. The virus is stained red using the antibody against PDCoV-N. The nucleus is stained blue (Scale bar = 100 μm). **(B)** Comparison of PDCoV replication levels in IPEC-J2 and monolayer PIEs by RT-qPCR. The experiment was repeated thrice and the data are shown as mean ± SD. **(C)** Transcriptional levels of genes indicated above in Enteroids and IPEC-J2 after 24 h of infection with PDCoV using RT-qPCR. Data were normalized to mock-infected controls (^∗^*p* < 0.05 and ^∗∗^*p* < 0.01).

IPEC-J2 cell is a conventional *in vitro* infection model to study the mechanisms of interaction between the host and pathogens (Zhao et al., 2014). However, there is little information on the immune responses of MPIEs infected by PDCoV. In this study, transcription of the immune-related genes was evaluated in MPIEs and IPEC-J2 cells. Although PDCoV could infect both MPIEs and IPEC-J2 and had a similar replicating ability (Figures 6A,B), expression of mucosal immune genes *Mucin2* and *MMP9*, as well as other innate immune genes *Stat1/3*, *OAS2*, *HERC5*, *ISG15*, and *CXCL11*, was upregulated significantly in MPIEs at 24 hpi (*p* < 0.05) compared to that in IPEC-J2 cells (Figure 6C). This shows that MPIE is a better choice for *in vitro* study as a PDCoV infection model than the commonly used IPEC-J2 cell line.

## Discussion

This study indicates that a porcine intestinal enteroids model was successfully developed as a “mini gut” suitable to study cell responses to PDCoV infection. The culture method we established is relatively easy without the necessity of cell-sorting steps. The quantity and purity of the separated crypts are high. In our culture method, the cultured PIEs are of almost 100% viability. A previous study has shown that the crypts from duodenum are relatively deeper compared with those from jejunum and ileum (Chwen et al., 2013). This suggests that duodenum might have better renewal frequency and potential than other intestinal segments. Duodenum was reported as having the best *in vitro* culture efficiency (but without presentation of experimental data) (Zietek et al., 2015). Our results support the conclusion that duodenum is superior to other segments probably as a result of expression of some specific genes in duodenum. A previous study has found different expression levels of the same genes located in different cells, although all cells are exposed to the same cytokines (Tsai et al., 2018). In another study, transcription of genes specific in some intestinal segments was not affected by cell passaging, and this special expression patterns could maintain for 10 to 12 weeks. However, most reported DEGs (differentially expressed genes) are related to nutrition absorption and transportation, such as GLP-1 and GIP (Middendorp et al., 2014). Evidence for the DEGs related to renewal is still lacking.

The clone forming efficiency was greater than 100% probably because the single living cell was not considered as an enteroid on the first day of culture. In long-term epithelial cell cultures, enteroids from duodenum, jejunum, and ileum still have specific characteristics, even though all the cells are exposed to the same extracellular factors (Middendorp et al., 2014). Similar to previous studies, the enteroids we cultured could live normally for 4 months with no anomalies in their growth, morphology, and pathogen infection rates during culture. Some studies reported that the enteroids could be cultured for more than 1 year with very little changes in characteristics as shown by single-cell sequencing (Fujii et al., 2018). Thus, this kind of enteroid could be employed for further studies on different biological functions, as it has similar biological characteristics.

The study of 3-D enteroids provides new inspiration for intestinal biology, but the relatively closed three-dimensional structure is an important practical limitation for biomedical research. For example, it is hard to perform quantitative analysis and viral infection, and it can also cause severe damage of enteroids proceeded micro-injection (Bartfeld and Clevers, 2015). Thorne et al. (2018) have established a simple 2-D enteroid monolayer culture system, which can recapitulate many properties of the intestinal epithelium and 3-D organoids. The MPIEs we cultured could form a tight confluent monolayer in 3 days and allow the virus to easily enter from the apical surface of the mucosa. This is also important for studying cell signaling and cellular responses, as many receptors are differentially expressed in the apical and baso-lateral membranes, including transporters, Toll-like receptors, and cytokine receptors (Playford et al., 1996; Kesisoglou et al., 2010; Grenier et al., 2013; Cevallos Porta et al., 2016). In addition, monolayer PIEs contain a repertoire of differentiated cell types and some progenitor cells. Human noroviruses (HuNoVs) can infect enterocytes (Ettayebi et al., 2016), rotaviruses can infect enterocytes and enteroendocrine cells (Saxena et al., 2016), and echovirus 11 preferentially infects enteroendocrine cells (Drummond et al., 2017). Our results indicate that PDCoV can replicate in the Sox9 + cells and Villin1-positive enterocytes. PDCoV has been found to locate mainly in the villi in the early stage of infection, but a small number could be present in crypts and Peyer’s patch during the late stage of infection (Hu et al., 2016). The spike (S) protein of coronaviruses functions as the key element for invasion into the cells by interaction with the receptor (Millet and Whittaker, 2015) and its activation is dependent on host cell proteases. This indicates that the stem cells and the absorptive enterocytes might possess the same or similar proteases. In short, MPIEs can be used as a system through which the enterovirus-gastrointestinal interactions can be simulated to reproduce the details of events associated with viral infections *in vivo*.

Viral infection could affect transcription of different cell marker genes. Our results show that the number of stem cells in the 2-D enteroids decreased after differentiation and transcription of *Lgr5* was not detectable (Figure 5D). However, PDCoV infection induced significant upregulation of *Bmi1* transcription, possibly due to the functions of intestinal stem cells (ISCs). So far, two ISC groups have been confirmed in the intestinal crypts, activated intestinal stem cells (aISCs) and reserved intestinal stem cells (rISCs). The gene markers of the aISCs include Lgr5, Ascl2, and Sox9. The rISCs are located in the “+4 to +6” position at the bottom of the crypts with BMI1 as the main marker. A previous study has shown that the number of the aISCs decreased significantly in the crypts exposed to radiation followed by the activation of rISCs for ISCs’ regeneration (Kim et al., 2017). Similarly, rISCs might be activated for intestine repair after PDCoV infection. Ki67, which is related to the cell proliferation and injury repair, was upregulated in the early stage of the intestinal injury in the ischemia–reperfusion (I/R) model (Abdeen et al., 2011). Thus, injury of the ISCs might be the reason why *Ki67* was upregulated in the early stage of PDCoV infection in our study.

Globet cell is a member of the Trefoil factor family (TFF) responsible for the reconstruction of cell surface continuity by promoting epithelial cells to migrate along the basal lamina (Kalabis et al., 2006). This biological function of TFF3 is not related to stem cell proliferation. Chromogranin A is reported to function as the precursor to several functional peptides, including pancreastatin (Zhang et al., 2006). Trypsin is required in the cultured cells for PDCoV infection (Jung et al., 2018). We suppose that upregulation of *ChgA* in PDCoV-infected cells might be related to the suppression of viral replication.

Our research also showed that PDCoV could infect enteroids, resulting in significant up-regulation of the +4 stem cell marker gene *Bmi1*. A previous study reported that the *Bmi1*-expressing stem cells are activated to replenish the Lgr5-expressing cells under pathological conditions (Tian et al., 2011). This could explain why the stem cell marker gene *Lgr5* was not detectable by RT-qPCR at 24 hpi (Figure 5D). Therefore, up-regulation of *Bmi1* indicates differentiation of large amount of +4 stem cells in response to PDCoV infection. Up-regulation of the genes related to markers of the secretory cells, such as *ChgA*, *Lyz*, and *TFF3*, may be due to a protective mechanism of the host cells by secreting mucus, antibacterial peptides, and other substances (Gill et al., 2011). We also found that the PDCoV infection can induce a much stronger immune response of enteroids than that of IPEC-J2 cells, which may be due to the presence of more stem cells in MPIEs (Andersson-Rolf et al., 2017).

In conclusion, this study indicates that 2-D enteroids derived from porcine jejunum are susceptible to PDCoV infection and the Sox9 + cells and villin1-positive enterocytes could be the target cells of PDCoV infection. This 2-D enteroids model should be superior to commonly used IPEC-J2 cells for investigation of immune responses of intestinal cells to infection by porcine enteric pathogens, such as PDCoV. We anticipate that enteroid infection models will play an important role in the study of enteroviruses replication and pathogenesis.

## Data Availability Statement

The datasets generated for this study are available on request to the corresponding author.

## Ethics Statement

The animal study was reviewed and approved by the Animal Care and Use Committee of Zhejiang University.

## Author Contributions

All authors listed have made a substantial, direct and intellectual contribution to the work, and approved it for publication.

## Conflict of Interest

The authors declare that the research was conducted in the absence of any commercial or financial relationships that could be construed as a potential conflict of interest.
